# Complete Genome Sequence of Hafnia paralvei Isolate AVS0177, Harboring *mcr-9* on a Plasmid

**DOI:** 10.1128/MRA.00966-21

**Published:** 2022-01-06

**Authors:** Michael Biggel, Katrin Zurfluh, Sarah Hoehn, Kira Schmitt, Andrea Frei, Christoph Jans, Roger Stephan

**Affiliations:** a Institute for Food Safety and Hygiene, Vetsuisse Faculty University of Zürich, Zürich, Switzerland; b Amt für Verbraucherschutz, Kanton Zug, Steinhausen, Switzerland; Loyola University Chicago

## Abstract

Here, we report the complete genome sequence of a Hafnia paralvei strain isolated from a lake in Switzerland in 2020. The genome consists of a 4.7-Mbp chromosome, a large plasmid (213 kb) harboring *mcr-9*, and a small plasmid (6 kb).

## ANNOUNCEMENT

Since the initial recognition of *Enterobacteriaceae* strains harboring the plasmid-mediated colistin resistance gene *mcr-1* in 2015 (1), nine additional *mcr* variants (*mcr-2* to *mcr-10*) have been described in various species (2–4). Here, we describe the occurrence of *mcr-9* in a *Hafnia* isolate, an enterobacterial genus naturally resistant to colistin (5).

AVS0177, showing phenotypical resistance to colistin (MIC = 8 mg/mL) and a positive PCR result for *mcr-9*, was isolated in November 2020 from a water sample collected at a depth of 120 m in Lake Zug, Switzerland. The water sample (100 mL) was filtered through a 0.45-μm membrane filter (Millipore). The filter was incubated in 10 mL *Enterobacteriaceae* enrichment (EE) broth (BD) at 37°C for 24 h. One loopful of the enrichment broth was spread onto cystine lactose electrolyte-deficient (CLED) agar (Oxoid) supplemented with 4 mg/mL colistin, 10 mg/mL vancomycin, and 5 mg/mL amphotericin and incubated at 37°C for 24 h. Matrix-assisted laser desorption ionization–time of flight mass spectrometry (MALDI-TOF MS) (Bruker Daltonics) was used for preliminary genus identification. Different PCR primers were used to detect the genes *mcr-1* to *mcr-10* (6–8). Colistin susceptibility testing was performed by broth dilution and interpreted according to EUCAST v11.0 protocols (https://www.eucast.org/clinical_breakpoints/). DNA was isolated from a subculture obtained from a single colony grown for 24 h at 37°C on sheep blood agar. For short-read sequencing, DNA was extracted using the DNeasy blood and tissue kit (Qiagen). Libraries were prepared using the Nextera DNA Flex library preparation kit (Illumina) and sequenced on the Illumina MiniSeq platform (2 × 150 bp). For long-read sequencing, DNA was obtained using the MasterPure Complete DNA and RNA purification kit (Lucigen) (no size selection/shearing). Multiplex libraries were prepared using the SQK-LSK109 kit with the EXP-NBD114 barcodes and sequenced using a MinION device on a FLO-MIN106 flow cell (Oxford Nanopore Technologies). Base calling was performed using Guppy CPU v4.2.2+effbaf8. Illumina and ONT adapters were trimmed using Trim Galore v0.6.6 (9) and Porechop v0.2.4 (10) and quality assessed using FastQC v0.11.9 (https://www.bioinformatics.babraham.ac.uk/projects/fastqc/) and LongQC v1.2.0 (11), respectively. A hybrid assembly was generated from 290 Mbp long-read (16,912 reads; read *N*_50_, 45 kb; coverage, 59×) and 222 Mbp short-read (754,150 paired-end reads; coverage, 45×) data using the Unicycler v0.4.8 pipeline (12), which includes assembly polishing, circularization, and rotation. The assembly was annotated using Prokaryotic Genome Annotation Pipeline (PGAP) (13). Resistance genes and plasmid replicons were identified using ABRicate v1.0.1 (14) (coverage/identity, >70%/>90%) with the ResFinder (15) and PlasmidFinder (16) databases, respectively. Default parameters were used for all software unless otherwise specified.

The complete genome of AVS0177 consisted of a circular 4,727,161-bp chromosome (rotated to start at *dnaA*) and the circular plasmids pAVS0177-a (212,640 bp) and pAVS0177-b (5,733 bp), with a GC content of 48.07%. rMLST analysis (17) assigned AVS0177 to the species Hafnia paralvei. Two antimicrobial resistance genes were detected: the chromosomally encoded *bla*_ACC-1a_ (AmpC beta-lactamase) and *mcr-9*, located on plasmid pAVS0177-a. pAVS0177-a also harbored the *fecABCDE* gene cluster, encoding a ferric citrate transport system (18), and the *sil* gene cluster, encoding copper and silver resistance (19, 20). The incompatibility (Inc) type of pAVS0177-a could not be identified, and no similar plasmids (i.e., query coverage of >50%) were found in a BLASTn (MegaBLAST) search of the NCBI nucleotide collection. The 12.5-kb genetic region encompassing *mcr-9* (spanning from the transcriptional repressor *rncR* to the insertion sequence IS*15DII* and including IS*903*B directly upstream of *mcr-9*) was structurally identical to the genetic context of *mcr-9* described in other species (21, 22). In conclusion, we present the complete genome sequence of an *mcr*-harboring *Hafnia* isolate and demonstrate that intrinsically resistant species may serve as reservoirs for transferable *mcr* genes.

### Data availability.

The complete genome sequence of AVS0177 has been deposited in GenBank under the accession numbers CP083737.1 (chromosome), CP083738.1 (pAVS0177-a), and CP083739.1 (pAVS0177-b) and the BioProject accession number PRJNA761908. The raw data were deposited in the NCBI Sequence Read Archive (SRA) under the accession numbers SRR15833986 (ONT reads) and SRR15833987 (Illumina reads).
